# Effects of potassium channel openers in the isolated perfused hypokalaemic murine heart

**DOI:** 10.1111/j.1748-1716.2007.01773.x

**Published:** 2008-05-01

**Authors:** M J Killeen, G Thomas, S-P Olesen, J Demnitz, K S Stokoe, A A Grace, C L-H Huang

**Affiliations:** 1 Physiological Laboratory, University of Cambridge Cambridge, UK; 2 Section of Cardiovascular Biology, Department of Biochemistry, University of Cambridge Cambridge, UK; 3 NeuroSearch A/S Ballerup, Denmark; 4 Danish National Research Foundation Centre for Cardiac Arrhythmia, Department of Biomedical Sciences, University of Copenhagen Copenhagen, Denmark

**Keywords:** hypokalaemia, mouse heart, nicorandil, NS1643

## Abstract

**Aim:**

We explored the anti-arrhythmic efficacy of K^+^ channel activation in the hypokalaemic murine heart using NS1643 and nicorandil, compounds which augment *I*_Kr_ and *I*_KATP_ respectively.

**Methods:**

Left ventricular epicardial and endocardial monophasic action potentials were compared in normokalaemic and hypokalaemic preparations in the absence and presence of NS1643 (30 μm) and nicorandil (20 μm).

**Results:**

Spontaneously beating hypokalaemic hearts (3 mm K^+^) all elicited early afterdepolarizations (EADs) and episodes of ventricular tachycardia (VT). Perfusion with NS1643 and nicorandil suppressed EADs and VT in 7 of 13 and five of six hypokalaemic hearts. Provoked arrhythmia studies using programmed electrical stimulation induced VT in all hypokalaemic hearts, but failed to do so in 7 of 13 and five of six hearts perfused with NS1643 and nicorandil respectively. These anti-arrhythmic effects were accompanied by reductions in action potential duration at 90% repolarization (APD_90_) and changes in the transmural gradient of repolarization, reflected in ΔAPD_90_. NS1643 and nicorandil reduced epicardial APD_90_ from 68.3 ± 1.1 to 56.5 ± 4.1 and 51.5 ± 1.5 ms, respectively, but preserved endocardial APD_90_ in hypokalaemic hearts. NS1643 and nicorandil thus restored ΔAPD_90_ from −9.6 ± 4.3 ms under baseline hypokalaemic conditions to 3.9 ± 4.1 and 9.9 ± 2.1 ms, respectively, close to normokalaemic values.

**Conclusion:**

These findings demonstrate, for the first time, the anti-arrhythmic efficacy of K^+^ channel activation in the setting of hypokalaemia. NS1643 and nicorandil are anti-arrhythmic through the suppression of EADs, reductions in APD_90_ and restorations of ΔAPD_90_.

Cardiac arrhythmias are one of the leading causes of morbidity and mortality in the developed world, accounting for up to 70 000 deaths per year in the UK (NICE, 2000). Long QT syndrome (LQTS) is a disorder of impaired ventricular repolarization that predisposes individuals to the development of lethal arrhythmic episodes (Keating & Sanguinetti 2001): congenital LQTS is predominantly caused by loss-of-function mutations in HERG or KCNQ1 K^+^ channel genes (Keating & Sanguinetti 2001). However, inheritable arrhythmias comprising LQTS account for only 1–2% of lethal ventricular arrhythmias seen clinically (Kuo *et al.* 2001); thus other important risk factors such as acquired forms of LQTS (Roden 2004) and hypokalaemia (Berthet *et al.* 1999) also play an important role in the induction of arrhythmogenesis. We have recently developed an intact murine whole heart model of hypokalaemia-induced arrhythmogenesis that shows action potential prolongation and altered transmural gradients of repolarization alongside early afterdepolarizations (EADs) and ventricular tachycardia (VT) (Killeen *et al.* 2007a). At the single cell level, hypokalaemia significantly reduced transient outward (*I*_to_) and inward rectifier (*I*_K1_) K^+^ currents, effects which accounted for prolonged action potential durations (APD) reported at the whole heart level (Killeen *et al.* 2007a).

Improving cardiac repolarization through activation of K^+^ channels, therefore, could potentially negate lethal arrhythmic episodes encountered under congenital and acquired LQTS and in hypokalaemia by enhancing outward repolarizing K^+^ current. Such actions would be expected to reduce APD and electrocardiographic QT interval, which may act to reduce the occurrence of EADs, critical initiation factors in the pathogenesis of VT (Killeen *et al.* 2007a,b). Pharmacological activation of K^+^ channels is a potentially novel anti-arrhythmic mechanism of action for the treatment of life-threatening arrhythmias in the setting of hypokalaemia. Recently, a novel and specific activator of the human *ether-a-go-go* related gene (HERG) K^+^ channel, NS1643, has been reported (Hansen *et al.* 2006). NS1643 has been shown to enhance the magnitude of HERG current through reductions in channel inactivation (Casis *et al.* 2006), and to reduce APD in isolated ventricular myocytes (Hansen *et al.* 2006). Nicorandil is an activator of K_ATP_ channels that was previously used as an antianginal agent. More recently, however, reports have suggested that nicorandil is an effective anti-arrhythmic agent in the setting of LQTS (Shimizu *et al.* 1998, Shimizu & Antzelevitch 2000).

The purpose of this study was to characterize the anti-arrhythmic efficacy of HERG K^+^ channel activation by NS1643 and K_ATP_ K^+^ channel activation by nicorandil in the setting of the common clinical condition of hypokalaemia, using a recently developed murine whole heart model of hypokalaemia-induced arrhythmogenesis (Killeen *et al.* 2007a). First, we demonstrated that NS1643 and nicorandil reduce the occurrence of EADs and episodes of spontaneous, unprovoked VT in the hypokalaemic murine heart. Secondly, we quantified the anti-arrhythmic efficacy of NS1643 and nicorandil in the hypokalaemic murine heart by using an established clinical method of arrhythmia provocation. Premature stimuli successfully initiated VT in all hypokalaemic hearts, but failed to do so in 7 of 13 hearts treated with NS1643 and in five of six hearts treated with nicorandil. Finally, we report that NS1643 and nicorandil reduce epicardial APD in the murine heart, actions which restore the expected transmural gradient of repolarization in the hypokalaemic murine left ventricle. Collectively, these findings describe the anti-arrhythmic efficacy of HERG and K_ATP_ K^+^ channel activation in the setting of hypokalaemia for the first time in *any* cardiac preparation.

## Methods

### Experimental animals

The mice used in this study were kept in an animal house at room temperature and subjected to a consistent 12 h : 12 h light : dark cycle and fed sterile rodent chow, having access to water at all times. Wild-type (WT) 129 background male and female mice, aged 5–7 months, were used in all experiments.

### Langendorff-perfused preparation

The experiments used a Langendorff-perfused preparation that was previously adapted for murine hearts (Balasubramaniam *et al.* 2003). Briefly, mice were killed by cervical dislocation in accordance with Schedule 1 of the UK Animals (Scientific Procedures) Act 1986. The heart was then quickly excised and submerged in ice-cold bicarbonate-buffered Krebs-Henseleit solution containing in mm: 119 NaCl, 25 NaHCO_3_, 4 KCl, 1.2 KH_2_PO_4_, 1 MgCl_2_, 1.8 CaCl_2_, 10 glucose and 2 sodium pyruvate. The solution was bubbled with a 95% O_2_–5% CO_2_ mixture (British Oxygen Company, Manchester, UK). The aorta was cannulated under the buffer surface using a 21-gauge custom-made cannula, and was attached to the cannula needle using a micro aneurysm clip (Harvard Apparatus, Edenbridge, UK). The preparation was then transferred to the perfusion apparatus, to which the cannula was attached, and perfusion commenced in a retrograde manner via the aorta with the above-mentioned bicarbonate-buffered Krebs-Henseleit solution. Before entering the aorta, the buffer was passed through 200 and 5 μm filters (Millipore, Watford, UK) and warmed to 37 °C by means of a water jacket and circulator (model C-85A; Techne, Cambridge, UK). Perfusion was maintained at a constant flow rate of 2–2.5 mL min^−1^ using a peristaltic pump (Watson-Marlow Bredel pumps model 505S; Falmouth, Cornwall, UK). Following the start of perfusion, healthy, experimentally viable hearts regained a pink coloration and spontaneous rhythmic contraction with warming. In 10% of experiments, hearts were discarded because of signs of ischaemia after cannulation and perfusion.

### Perfused heart electrophysiological measurements

In the present experiments, a paired (1 mm inter-pole spacing) platinum-stimulating electrode was placed on the basal surface of the right ventricular epicardium. Prior to experimental procedures, hearts were paced for 10 min at 8 Hz using 2 ms square-wave stimuli with amplitudes set to three times the excitation threshold (Grass S48 stimulator; Grass-Telefactor, Slough, UK).

Epicardial monophasic action potential (MAP) recordings were obtained using a MAP electrode (Linton Instruments, Harvard Apparatus) placed on the basal surface of the left ventricular epicardium. The epicardial MAP electrode was gradually positioned until a gentle but stable contact pressure was achieved. This resulted in a recording of MAP signals. For endocardial recordings, a small access window was created in the interventricular septum to gain access to the left ventricular endocardium (Casimiro *et al.* 2001). A custom-made endocardial MAP electrode constructed from two twisted strands of Teflon-coated (0.25 mm diameter) silver wire (99.99% purity) (Advent Research Materials Ltd, Oxford, UK) that had been previously galvanically chlorided to eliminate DC offset was positioned on to the left ventricular free wall under a stable contact pressure until MAP signals were achieved. MAPs were amplified, band-pass filtered (30 Hz to 1 kHz: Gould 2400S; Gould-Nicolet Technologies, Ilford, Essex, UK) and digitized (1401 plus MKII; Cambridge Electronic Design, Cambridge, UK). MAPs were extracted and analysed (Spike II version 4: Cambridge Electronic Design) to derive the precise duration of the digitized signals. The recordings were deemed reproducible and, hence of an acceptable standard for analysis, if they had the following properties: a stable baseline, a rapid upstroke phase with consistent amplitude, a smooth contoured repolarization phase and a stable duration (MAP duration at 90% repolarization (APD_90_) was reproducible within 3 ms under baseline conditions).

### Experimental protocol

A standard pacing protocol (basic cycle length, BCL of 125 ms) that corresponded to physiological whole animal heart rates (Papadatos *et al.* 2002) was initiated for periods of up to 20 min to measure APD at 50%, 70% and 90% repolarization. External pacing stimuli were subsequently withdrawn from all preparations, leading to a significantly reduced, intrinsic heart rate corresponding to a BCL of approximately 400 ms. Reduced heart rates are a known risk factor for the development of repolarization abnormalities such as EADs and triggered beats that may underlie the induction of VT (Roden & Hoffman 1985). Epicardial MAPs were recorded for up to 20 min from isolated, perfused WT mouse hearts under intrinsic pacing conditions. Following this, programmed electrical stimulation (PES) of the heart was carried out using an adaptation of the corresponding clinical techniques (Saumarez & Grace 2000, Balasubramaniam *et al.* 2003). PES procedures began by applying standard pacing stimuli at a BCL of 125 ms for 25 s. Following this, a drive train of eight paced beats (S1) again at a BCL of 125 ms preceded an extrastimulus (S2) every ninth beat. S1S2 intervals initially equalled the pacing interval and then were progressively reduced by 1 ms with each nine beat cycle until ventricular refractoriness was reached, at which point the S2 stimulus elicited no MAP. Recordings were subsequently repeated following a 20-min wash-in of a reduced [K^+^]_o_ perfusate, of 3 mm in the absence and presence of NS1643 (30 μm) or nicorandil (20 μm).

To quantify changes in transmural gradients of repolarization, ΔAPD_90_ was calculated from the difference between the mean endocardial and epicardial APD_90_ values, giving positive results where the endocardial value exceeded the epicardial value, and negative results where the epicardial value was greater. An EAD was defined as a positive deflection that interrupted the smooth repolarization phase of the AP. A triggered beat was similarly described as a positive deflection in the smooth repolarization phase of the action potential whose amplitude approximately matched the amplitude of the initial action potential. Arrhythmias were defined as ventricular tachyarrhythmias of more than five cycles in duration that were typically self-terminating. Following cannulation and subsequent perfusion of hearts, approximately 10% of preparations were discarded because of signs of ischaemia. Preliminary experiments revealed that there were no gender-related effects in the response to either hypokalaemia, NS1643 or nicorandil. These findings are in keeping with our earlier studies using the hypokalaemic murine heart (Killeen *et al.* 2007a,b) and the genetically modified Brugada syndrome and Long QT 3 syndrome mouse models (Stokoe *et al.* 2007a,b, Thomas *et al.* 2007a,b), which similarly revealed no gender-related effects.

### Experimental solutions

NS1643 was synthesized by the Department of Chemistry at NeuroSearch (Ballerup, Denmark). The drug was initially prepared as a 100 mm stock solution in dimethly sulphoxide (DMSO) and stored at −20 °C. Nicorandil was purchased from Tocris Limited (Bristol, UK) and was initially prepared as a 10 mm stock solution in distilled water and stored at −20°C. Subsequent dilutions of both drugs were made in the perfusion buffer.

### Statistical analysis

MAP data were initially imported into Microsoft excel. All data are expressed as mean ± SEM. Comparisons were made using anova (spss software) with *P* < 0.05 being considered significant.

## Results

### Effects of NS1643 and nicorandil on arrhythmogenesis in spontaneously beating hypokalaemic hearts

Following cannulation and perfusion the electrophysiological parameters of MAP waveform morphology, amplitude and duration reached a steady state within 10 min. MAP recordings in murine hearts paced at a basic cycle length (BCL) of 125 ms subsequently remained highly reproducible throughout the duration of all experiments.

Extrinsic pacing was then terminated, resulting in a reduced heart rate (180 beats per minute compared to 480 beats per minute under extrinsic pacing at a BCL of 125 ms). Bradycardia is a recognized risk factor for the development of torsade de pointes (Roden & Hoffman 1985) and earlier studies have reported a higher prevalence of EADs and associated VT under bradycardic conditions (Milberg *et al.* 2002, Fabritz *et al.* 2003, Kirchhof *et al.* 2004, Killeen *et al.* 2007a,b).

**Figure 1:**
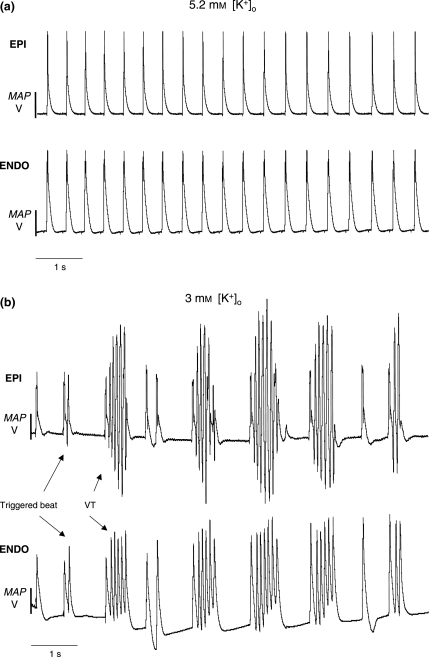
Representative trace of simultaneously recorded left ventricular epicardial and endocardial MAPs from an isolated, spontaneously beating, Langendorff-perfused (a) normokalaemic (5.2 mm [K^+^]_o_) and (b) hypokalaemic (3 mm [K^+^]_o_) murine heart in the absence of extrinsic pacing. Under normokalaemic conditions, no repolarization abnormalities or episodes of ventricular tachycardia (VT) were ever recorded from any preparation (*n* = 6). Following perfusion with hypokalaemic solutions, early afterdepolarizations and triggered beats that preceded episodes of VT were recorded in all preparations from both epicardial *and* endocardial surfaces (*n* = 6).

Under control normokalaemic conditions, both epicardial and endocardial waveforms showed no repolarization abnormalities and no episodes of VT (*n* = 6) (Fig. 1a). However, following the reduction in [K^+^]_o_ from 5.2 to 3 mm, EADs and triggered beats followed by episodes of non-sustained VT were observed from all hypokalaemic preparations, from both left ventricular epicardial *and* endocardial surfaces (*n* = 6) (Fig. 1b).

**Figure 2:**
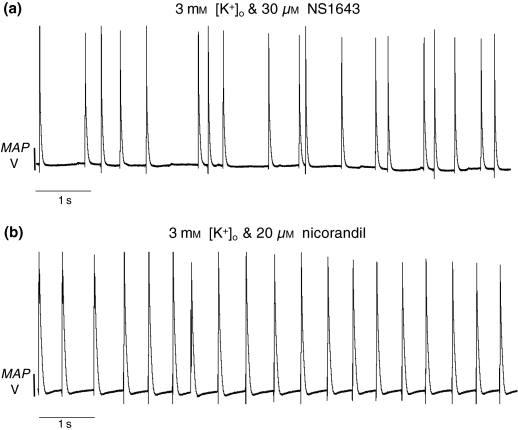
Representative left ventricular epicardial MAPs recorded from a spontaneously beating hypokalaemic heart in the presence of (a) 30 μm NS1643 and (b) 20 μm nicorandil. Perfusion of hypokalaemic hearts with NS1643 or nicorandil eliminated EADs, triggered beats and episodes of unprovoked arrhythmogenesis in 7 of 13 and five of six preparations respectively.

We subsequently tested the anti-arrhythmic efficacy of NS1643 in the suppression of spontaneously occurring, unprovoked arrhythmogenesis in the hypokalaemic murine heart. Following continued perfusion for 20 min, whilst there was a persistence of closely coupled beats in all the traces obtained, NS1643 suppressed all repolarization abnormalities and spontaneous arrhythmias in epicardial recordings from 7 of 13 (54%) hypokalaemic preparations (Fig. 2a).

In preparations perfused instead with nicorandil (20 μm) for 20 min, neither EADs nor episodes of unprovoked spontaneous arrhythmias were seen in five of six (83%) hypokalaemic preparations (Fig. 2b). We then proceeded to investigate the basis of these anti-arrhythmic effects of NS1643 and nicorandil in the hypokalaemic murine hearts using established arrhythmia provocation protocols.

### Effects of NS1643 and nicorandil on provoked arrhythmogenesis in hypokalaemic hearts

**Figure 3:**
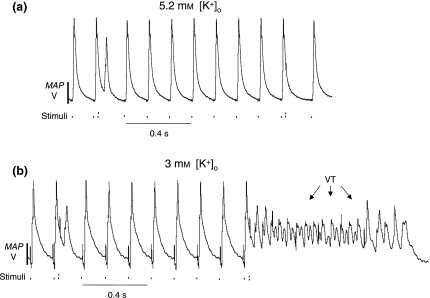
Programmed electrical stimulation (PES) of isolated, wild-type Langendorff-perfused mouse hearts under (a) control normokalaemic conditions and (b) following perfusion with 3 mm [K^+^]_o_ hypokalaemic buffer solutions. PES failed to induce ventricular tachycardia (VT) in any preparation perfused with control, normokalaemic buffer (*n* = 6). However, PES induced VT in six of six hearts perfused with 3 mm [K^+^]_o_.

**Figure 4:**
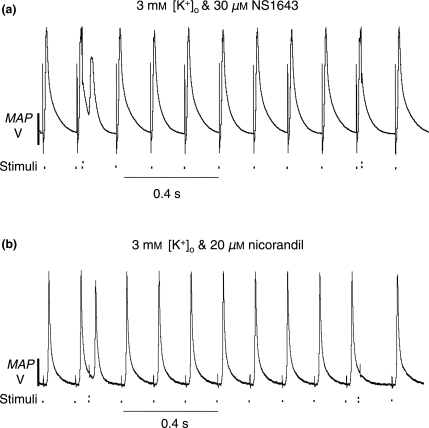
Programmed electrical stimulation (PES) in isolated, Langendorff-perfused hypokalaemic murine hearts following perfusion with (a) 30 μm NS1643 and (b) 20 μm nicorandil. In the presence of NS1643 or nicorandil, PES failed to induce ventricular tachycardia in 7 of 13 and five of six preparations respectively.

Programmed electrical stimulation was used to determine the arrhythmic susceptibility produced by extrasystolic stimulation of hypokalaemic isolated murine hearts perfused with either 30 μm NS1643 or 20 μm nicorandil. These standard PES procedures resembled clinical diagnostic techniques used to assess arrhythmogenic propensity in patients for the current murine whole heart model at a pacing frequency, which closely corresponded to the *in vivo* murine heart rate (Saumarez & Grace 2000, Balasubramaniam *et al.* 2003, Yang *et al.* 2007). Extrasystolic (S2) stimuli mimicked EADs, however, producing a significantly larger stimulus than a spontaneously occurring EAD. Short S1-S2 coupling intervals elicited typical extrasystolic MAPs in hearts in both normokalaemic and hypokalaemic conditions (Fig. 3). In all preparations perfused with control normokalaemic solutions, PES failed to induce VT (Fig. 3a). In contrast, closely coupled extra stimuli successfully and reproducibly induced non-sustained VT in six of six hypokalaemic preparations (Fig. 3b). In contrast, following perfusion with 30 μm NS1643 for 20 min, PES failed to induce VT with a persistence of normal regular rhythm in 7 of 13 (54%) hypokalaemic hearts (Fig. 4a). In parallel with this, in hypokalaemic hearts perfused with 20 μm nicorandil, PES failed to induce VT in five of six hearts (83%) (Fig. 4b).

NS1643 and nicorandil thus exerted an anti-arrhythmic effect in both the spontaneously beating and the provoked hypokalaemic murine heart. We hypothesized that these observed anti-arrhythmic effects may be associated with changes in APD and the left ventricular transmural gradient of repolarization.

### Effects of NS1643 and nicorandil on APD and transmural gradients of repolarization in hypokalaemic hearts

We therefore proceeded to examine the effects of hypokalaemia and perfusion with either NS1643 or nicorandil on APDs recorded from the left ventricular epicardial and endocardial surfaces to assess whether the anti-arrhythmic effects of NS1643 and nicorandil observed in unprovoked, spontaneously beating and provoked hearts correlated with transmural APD changes. Measurement of epicardial and endocardial APD permitted the quantification of the left ventricular transmural gradient of repolarization. We have recently correlated changes in ventricular repolarization gradients with arrhythmogenicity in a range of whole heart murine models (Killeen *et al.* 2007a,b,Stokoe *et al.* 2007a,b, Thomas *et al.* 2007a,b). We accordingly paced all preparations at a BCL of 125 ms, in keeping with previous studies (Killeen *et al.* 2007a,b).

**Figure 5:**
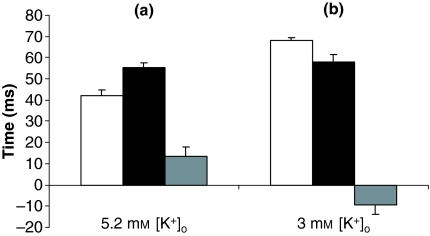
Steady-state epicardial and endocardial action potential duration (APD) measured at 90% repolarization (APD_90_), and ΔAPD_90_ values (white, black and grey columns respectively) under (a) control conditions (six hearts) and (b) following perfusion with 3 mm [K^+^]_o_ hypokalaemic solutions (*n* = 6). Hypokalaemia prolonged epicardial and endocardial APD and significantly changed the left ventricular transmural gradient of repolarization, ΔAPD_90_, to negative values.

Under normokalaemic conditions at a BCL of 125 ms, epicardial and endocardial APD_90_ were 41.9 ± 2.8 and 55.5 ± 2.0 ms respectively (*n* = 6) (Fig. 5a). The transmural gradient of repolarization, reflected as ΔAPD_90_, was 13.6 ± 4.1 ms (*n* = 6). Perfusion of normokalaemic hearts with 30 μm NS1643 produced no significant effect upon epicardial or endocardial APD_90_ (47.1 ± 0.7 and 48.5 ± 4.34 ms, respectively), and ΔAPD_90_ was reduced to 1.4 ± 4.4 ms (*n* = 6, *P* < 0.05). This reduction in ΔAPD_90_ following perfusion of normokalaemic hearts with 30 μm NS1643 was not associated with arrhythmogenicity in any spontaneously beating or provoked heart (*n* = 6). Perfusion of normokalaemic hearts with 20 μm nicorandil significantly reduced epicardial APD_90_ to 31.3 ± 1.1 ms (*n* = 6, *P* < 0.05) yet had no effect upon endocardial APD_90_ (56.4 ± 2.7 ms) (*n* = 6, *P* > 0.05). These effects significantly increased ΔAPD_90_ to 25.1 ± 2.9 ms (*n* = 6, *P* < 0.05); however, perfusion of normokalaemic hearts with 20 μm nicorandil did not induce any arrhythmic events in any spontaneously beating or provoked heart (*n* = 6).

**Figure 6:**
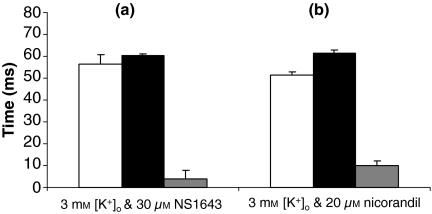
Steady-state epicardial and endocardial action potential duration (APD) measured at 90% repolarization (APD_90_), and ΔAPD_90_ values (white, black and grey columns respectively) under hypokalaemic conditions following perfusion with (a) 30 μm NS1643 (*n* = 13) or (b) 20 μm nicorandil (*n* = 6). Both NS1643 and nicorandil reduced epicardial APD and restored the left ventricular transmural gradient of repolarization, ΔAPD_90_, to positive values.

Perfusion of murine hearts with 3 mm [K^+^]_o_ led to significant epicardial and endocardial action potential prolongation. Epicardial APD_90_ was increased from 41.9 ± 2.8 to 68.3 ± 1.1 ms (*n* = 6, *P* < 0.05) (Fig. 5b). Hypokalaemia produced an appreciable but not significant increase in endocardial APD_90_ from 55.5 ± 2.0 ms to 58.2 ± 3.3 ms (*n* = 6, *P* > 0.05) (Fig. 5b). These changes in epicardial and endocardial APD significantly reduced ΔAPD_90_ from 13.6 ± 4.1 to −9.6 ± 4.3 ms (*n* = 6, *P* < 0.05) (Fig. 5b), in keeping with our previous findings (Killeen *et al.* 2007a,b). Administration of 30 μm NS1643 to hypokalaemic preparations produced an appreciable, but not significant, reduction in epicardial APD_90_ from 68.3 ± 1.1 to 56.5 ± 4.1 ms (*n* = 13, *P* > 0.05), whilst having no effect on endocardial APD_90_ (58.2 ± 3.3 ms vs. 60.4 ±0.8 ms, respectively, *n* = 13, *P* > 0.05) (Fig. 6a). Subsequently, perfusion of hypokalaemic hearts with 30 μm NS1643 significantly normalized the left ventricular transmural gradient of repolarization, reflected in ΔAPD_90_, to 3.9 ± 4.1 ms (*n* = 13, *P* < 0.05) (Fig. 6a).

Perfusion of hypokalaemic hearts with 20 μm nicorandil similarly produced changes in APD_90_ and ΔAPD_90_. Nicorandil significantly reduced epicardial APD_90_ to 51.5 ± 1.5 ms (*n* = 6, *P* < 0.05) whilst having no effect upon endocardial APD_90_ (61.4 ± 1.5 ms vs. 58.2 ± 3.3 ms respectively) (*n* = 6, *P* < 0.05) (Fig. 6b). These effects of selective, significant reduction in epicardial over endocardial APD following perfusion with 20 μm nicorandil in the hypokalaemic murine heart significantly normalized ΔAPD_90_ to values statistically indistinguishable from control normokalaemic values (9.9 ± 2.1 ms vs. 13.6 ± 4.1 ms respectively) (*n* = 12, *P* < 0.05) (Fig. 6b).

Perfusion of hypokalaemic hearts with NS1643 and nicorandil thus produced anti-arrhythmic effects through the suppression of EADs and triggered activity, epicardial APD reduction and the correction of altered transmural gradients of repolarization. These findings demonstrate that improving myocardial repolarization through the pharmacological activation of K^+^ channels is an effective anti-arrhythmic approach in the setting of hypokalaemia.

## Discussion

Hypokalaemia is known to predispose to cardiac arrhythmias at the clinical (Berthet *et al.* 1999) and experimental (Killeen *et al.* 2007a) levels through the prolongation of APD and the subsequent development of EADs, which superimpose upon an arrhythmogenic substrate of altered transmural gradients of repolarization (Killeen *et al.* 2007b). At the single cell level, we previously showed that hypokalaemia reduces transient outward current (*I*_to_) and inwardly rectifying current (*I*_K1_), effects that are likely to be responsible for APD prolongation reported at the whole heart level (Killeen *et al.* 2007a). Thus, improving myocardial repolarization through the pharmacological activation of K^+^ channels is an approach that would be expected to reduce APD and therefore reduce the incidence of EADs and of subsequent arrhythmogenesis.

We used a recently reported intact murine model of hypokalaemia-induced arrhythmogenesis (Killeen *et al.* 2007a) to explore, for the first time, the anti-arrhythmic efficacy of K^+^ channel activation in the setting of hypokalaemia. We report that pharmacological activation of both HERG and K_ATP_ K^+^ channels by the novel compound NS1643 and nicorandil respectively, is an effective anti-arrhythmic approach in the setting of hypokalaemia. In the hypokalaemic murine heart, NS1643 and nicorandil exert these anti-arrhythmic effects through a suppression of EADs and VT, reductions in epicardial APD, and a normalization of transmural gradients of repolarization. We used NS1643 and nicorandil at concentrations that exceeded their respective EC_50_ values to produce a marked electrophysiological response. Thus, NS1643 was perfused at a concentration of 30 μm (EC_50_ = 10.5 ±1.5 μm) (Hansen *et al.* 2006) and nicorandil was used at a concentration of 20 μm (EC_50_ = 10 μm) (Shindo *et al.* 1998).

First, in this study, we have shown that perfusion with NS1643 and nicorandil eliminates EADs, triggered beats and VT in 7 of 13 (54%) and five of six (83%) spontaneously beating hypokalaemic hearts respectively. EADs are critical initiation factors for lethal arrhythmias in the setting of prolonged APDs (Killeen *et al.* 2007a,b; Thomas *et al.* 2007a,b) and are believed to be caused by recovery of the L-type calcium channels (LTCCs) from inactivation, leading to a secondary influx of Ca^2+^ (January & Riddle 1989). We have recently shown that in the setting of hypokalaemia, selective inhibition of LTCCs and inhibition of the LTCC regulator, calmodulin kinase type II reduces EADs, triggered beats and episodes of spontaneous arrhythmogenesis associated with hypokalaemia (Killeen *et al.* 2007b). Augmenting outward repolarizing K^+^ currents would therefore be expected to restore any imbalance between inward Ca^2+^and outward K^+^ currents, acting to impair the formation of EADs and consequent arrhythmogenesis. In this study, we show that pharmacological activation of HERG and K_ATP_ K^+^ channels by NS1643 and nicorandil, respectively, reduced the occurrence of EADs, triggered beats and episodes of VT in the spontaneously beating hypokalaemic murine heart.

Secondly, we quantified the anti-arrhythmic efficacy of NS1643 and nicorandil in the hypokalaemic murine heart by using the established arrhythmia provocation protocol of PES. Premature stimuli applied during PES reliably induced VT in all hypokalaemic preparations, but failed to do so in 7 of 13 (54%) hypokalaemic hearts perfused with NS1643 and in five of six (83%) hypokalaemic hearts perfused with nicorandil.

Finally, we determined whether the anti-arrhythmic effects of NS1643 and nicorandil were caused by changes in epicardial and endocardial APD and transmural gradients of repolarization. We have recently correlated alterations in the transmural gradient of repolarization with arrhythmogenicity provoked by imposed extrasystolic stimuli at the whole heart level in the setting of hypokalaemia (Killeen *et al.* 2007a,b), and the LQTS type 3 (Stokoe *et al.* 2007a, Thomas *et al.* 2007a) and type 5 (Thomas *et al.* 2007b) in genetically modified murine hearts.

In this study, perfusion of hypokalaemic hearts with NS1643 and nicorandil reduced epicardial APD_90_, effects that led to the normalization of the murine left ventricular transmural gradient of APD. We have recently reported similar findings using the dihydropiridine L-type Ca^2+^ channel (LTCC) blocker nifedipine in hypokalaemic murine hearts (Killeen *et al.* 2007b) and in genetically modified murine hearts following targeted disruption of the KCNE1 gene to model human LQTS type 5 (Thomas *et al.* 2007b). In these studies, nifedipine reduced epicardial APD_90,_ which acted to correct the altered transmural gradient of repolarization in the hypokalaemic (Killeen *et al.* 2007b) and KCNE1 (Thomas *et al.* 2007b) murine hearts, actions that also significantly reduced arrhythmogenicity. Similarly, in this study, activation of HERG and K_ATP_ K^+^ channels, by NS1643 and nicorandil respectively, in the hypokalaemic murine heart reduced epicardial APD_90_ which corrected the altered left ventricular transmural gradient of APD to values similar to those recorded from control, normokalaemic hearts.

NS1643 is a novel small molecule compound that activates HERG K^+^ channels, leading to a greater outward repolarizing *I*_Kr_ and a shortening of APD in isolated ventricular myocytes (Hansen *et al.* 2006). It was previously shown that HERG channel overexpression in rabbit cardiac myocytes reduces the susceptibility to EADs and shortens APD (Nuss *et al.* 1999). Although the predominant repolarizing K^+^ current in the adult mouse ventricle is *I*_to_ (Nerbonne *et al.* 2001), *I*_Kr_ does contribute to repolarization in the adult murine heart, albeit to a lesser degree than *I*_to_. Recent studies have shown that the ion channel proteins that constitute *I*_Kr_ (mouse ERG transcript, mERG) are abundantly expressed in the adult murine ventricle, and give rise to an isolated current at a density similar to that measured in other mammals (Liu *et al.* 2004). In ventricular myocytes isolated from WT mice, a delayed rectifier current with gating properties similar to *I*_Kr_ in other species, including inward rectification properties at positive potentials and sensitivity to E-4031, has been consistently reported (Babij *et al.* 1998, Liu *et al.* 2004). Furthermore, specific block of *I*_Kr_, using E-4031, produced AP prolongation in transmembrane action potentials (TAP) from isolated adult murine ventricular myocytes (Babij *et al.* 1998) and in the intact murine ventricle (Charpentier *et al.* 1998). Finally, in the isolated, perfused murine whole heart, the *I*_Kr_ blocking agent sotalol significantly prolonged epicardial APD (Fabritz *et al.* 2003). Collectively, these findings suggest a role of *I*_Kr_ in murine ventricular repolarization. Despite the reduced role of *I*_Kr_ compared to *I*_to_ in murine cardiac repolarization, here we report, for the first time, that pharmacological activation of the HERG K^+^ channel suppresses spontaneously occurring EADs, triggered beats and episodes of VT at the whole heart level.

Nicorandil is an ATP-sensitive potassium (K_ATP_) channel opener that has been used extensively as an antianginal agent in many countries. K_ATP_ channels are members of the inward rectifier ion channel superfamily, and are present at high densities in cardiac myocytes (Morrissey *et al.* 2005). K_ATP_ channels are only weak inward rectifiers and, therefore, are capable of passing a substantial outward current (Shivkumar & Valderrabano 2004). A small number of reports have suggested that K_ATP_ channel openers are efficacious in the suppression of arrhythmogenesis in the setting of the LQTS (Shimizu *et al.* 1998, Shimizu & Antzelevitch 2000). Improving cardiac repolarization through increases in outward K^+^ current carried by pharmacologically activated K_ATP_ channels has been shown to eliminate EADs and episodes of arrhythmogenesis in LQT1 syndrome patients (Shimizu *et al.* 1998). In this study, we show that pharmacological activation of K_ATP_ K^+^ channels by nicorandil is an effective anti-arrhythmic approach in the common clinical condition of hypokalaemia.

A problem encountered with K_ATP_ channel openers is a fall in blood pressure and a reflex increase in sympathetic activity, which may be proarrhythmic. However, compared to other K_ATP_ channel openers such as pinacidil or chromokalim, nicorandil exerts only a mild hypotensive effect (Spinelli *et al.* 1990). Furthermore, all clinical case reports documenting the use of nicorandil for treating cardiac arrhythmias describe an *anti-arrhythmic* as opposed to a proarrhythmic effect of nicorandil (Chinushi *et al.* 1995); Shimizu *et al.* 1998). Indeed, in this study, under normokalaemic control conditions, nicorandil did not induce EADs, triggered beats or VT in any preparation and under hypokalaemic conditions, nicorandil was shown to be anti-arrhythmic.

Short QT syndrome (SQTS) is a recently described primary electrical disease of the heart, which is characterized by the presence of a QT interval less than 320 ms and a high incidence of ventricular arrhythmogenesis (Gussak & Antzelevitch 2000). Previously, studies genetically mapped SQTS to gain-of-function mutations in the genes encoding *I*_Kr_ (Brugada *et al.* 2004), *I*_Ks_ (Bellocq *et al.* 2004) and *I*_K1_ (Priori *et al.* 2005). These mutations give rise to larger repolarizing K^+^ currents and an expected abbreviation of APD and QT interval. Theoretically, pharmacological activation of *I*_Kr_ and *I*_KATP_ may mimic features of SQTS and could give rise to potentially lethal episodes of arrhythmia. However, we consider this unlikely for the following reasons.

First, a previously reported SQTS N588K gain-of-function mutation in HERG results in channels, which do not inactivate at physiological membrane potentials (Cordeiro *et al.* 2005). HERG channels treated with NS1643 behave in a very similar way to WT channels in the absence of NS1643: following depolarization, NS1643-activated HERG channels inactivate rapidly and recover from inactivation, passing an outward current as the membrane potential recovers (Hansen *et al.* 2006). These findings are in sharp contrast to mutant N588K channels, which monotonically followed the shape and amplitude of the action potential such that greater outward currents are observed in early phases of the AP (Cordeiro *et al.* 2005).

Secondly, the use of nicorandil to activate K_ATP_ channels and cause an increase in repolarizing current and a consequent shortening of APD and anti-arrhythmic effects in the setting of hypokalaemia is supported by the documented efficacy of this drug in the clinical setting (Shimizu *et al.* 1998). Activation of K_ATP_ channels was previously shown to reduce EADs and prevent episodes of arrhythmia in patients with congenital LQTS (Shimizu *et al.* 1998). Furthermore, at present, clinical trials of patients with ischaemic heart disease have revealed no findings of proarrhythmia associated with any K_ATP_ channel activators (Miyazaki *et al.* 1995, Markham *et al.* 2000). Finally, administration of nicorandil to patients with acute myocardial infarction at the time of coronary angioplasty was shown to reduce episodes of malignant ventricular arrhythmia (Ito *et al.* 1999).

Collectively, these findings suggest: (1) Pharmacological activation of HERG channels produces effects, which are not comparable to those observed with gain-of-function mutations in HERG comprising variants of SQTS. (2) Activation of K_ATP_ channels by nicorandil has been shown to be an effective anti-arrhythmic approach in the setting of impaired myocardial repolarization as revealed in the findings in this study and in clinical reports from congenital LQTS patients (Shimizu *et al.* 1998) in addition to more diverse cardiac diseases such as myocardial infarction (Ito *et al.* 1999). We therefore consider it unlikely that the use of NS1643 and nicorandil in conditions of compromised myocardial repolarization could give rise to an SQTS proarrhythmic phenotype.

This study demonstrates that activation of *I*_Kr_ and *I*_KATP_ is an effective pharmacological anti-arrhythmic approach in the setting of hypokalaemia. We previously demonstrated that hypokalaemia reduces *I*_to_ and *I*_K1_ (Killeen *et al.* 2007a) and other studies have reported reductions in *I*_Kr_ (Yang & Roden 1996). Activation of K^+^ channels, in situations in which repolarizing K^+^ currents are compromised, could be an important protective mechanism against lethal arrhythmias. In the setting of hypokalaemia, correction of serum K^+^ is a relatively safe and effective measure to prevent cardiac arrhythmias. However, this study acts as a proof of concept for the pharmacological activation of repolarizing K^+^ channels in conditions in which K^+^ channel function is reduced. Cardiac diseases comprising heart failure (Wang *et al.* 2006), congenital (Splawski *et al.* 2000) and acquired arrhythmia syndromes (Clancy *et al.* 2003) all have altered K^+^ channel function as a common prerequisite. This study, therefore, provides important information, which validates the anti-arrhythmic efficacy of K^+^ channel activation in response to acquired LQTS conditions, which could potentially compromise repolarizing K^+^ current function and give rise to lethal arrhythmias.

Taken together, these findings support the notion that increased repolarization through activation of HERG and K_ATP_ K^+^ channels is an important anti-arrhythmic mechanism of action by (1) suppression of EADs and triggered beats that are likely initiating factors for the development of VT; (2) reductions in myocardial repolarization times; and (3) normalization of transmural gradients of repolarization in the left ventricle. The use of established arrhythmia provocation protocols confirmed the anti-arrhythmic efficacy of NS1643 and nicorandil in the hypokalaemic murine heart. In this study, we demonstrate that nicorandil is more effective at reducing arrhythmogenicity in the hypokalaemic murine heart than NS1643. Both nicorandil and NS1643 were used at concentrations that exceeded their respective EC_50_ values to produce significant electrophysiological responses. In the murine heart, the predominant repolarizing K^+^ current is *I*_to_ (Nerbonne *et al.* 2001). *I*_Kr_, however, does play a role, albeit a lesser one than *I*_to_, in murine repolarization (Fabritz *et al.* 2003). NS1643 suppressed EADs alongside spontaneous VT in addition to protecting against provoked arrhythmias in 7 of 13 preparations in the setting of hypokalaemia. The reduced efficacy of NS1643 compared to nicorandil is likely to be due to the reduced role of *I*_Kr_ in murine ventricular repolarization. Nevertheless, this study shows for the first time in *any* cardiac preparation that activation of HERG and K_ATP_ K^+^ channels is an effective anti-arrhythmic approach in the murine heart in the setting of the common clinical condition of hypokalaemia, findings, which at the very least merit further testing.
